# Barriers and enablers of type 2 diabetes self‐management in people with severe mental illness

**DOI:** 10.1111/hex.12543

**Published:** 2017-03-17

**Authors:** Kathleen Mulligan, Hayley McBain, Frederique Lamontagne‐Godwin, Jacqui Chapman, Mark Haddad, Julia Jones, Chris Flood, David Thomas, Alan Simpson

**Affiliations:** ^1^ Centre for Health Services Research School of Health Sciences City, University of London London UK; ^2^ East London NHS Foundation Trust London UK; ^3^ Centre for Mental Health Research School of Health Sciences City, University of London London UK; ^4^ Centre for Research in Primary and Community Care (CRIPACC) School of Health and Social Work University of Hertfordshire Hatfield UK

**Keywords:** diabetes, self‐management, service users, severe mental illness, Theoretical Domains Framework

## Abstract

**Background:**

People with diabetes and severe mental illness (SMI) experience poorer outcomes than those with diabetes alone. To improve outcomes, it is necessary to understand the difficulties that people with SMI experience in managing their diabetes.

**Aims:**

To identify barriers and enablers to effective diabetes self‐management experienced by people with SMI and type 2 diabetes.

**Method:**

Qualitative methodology using semi‐structured interviews was employed. Development of the interview topic guide and analysis of the transcripts were informed by the Theoretical Domains Framework for behaviour change, which consists of fourteen theoretical domains that have been found to influence behaviour.

**Results:**

Fourteen people with SMI and type 2 diabetes took part in the study. Participants considered diabetes self‐management to be important, were aware of the risks of poor diabetes control but struggled to follow recommended advice, particularly if their mental health was poor. Support from family and health professionals was considered an important enabler of diabetes self‐management.

**Conclusions:**

New approaches are required to support diabetes self‐management in people with SMI. This study identified some of the important domains that may be targeted in new interventions.

## 1 INTRODUCTION

People with severe mental illnesses (SMI) such as schizophrenia and bipolar disorder experience health inequalities,^1^ among which is a twofold increased risk of type 2 diabetes compared with the general population.^2^, ^3^ Prevalence of diabetes in people with SMI is approximately 12%.^4^ Acute diabetes complications^5^ and mortality^6^ are greater in people who also have SMI, whilst the health‐related quality of life of people with SMI is poorer in those who also have diabetes.^7^

Effective control of type 2 diabetes requires performance of several self‐management tasks, which include taking medication, following a healthy diet, taking regular exercise, performing foot care and attending health checks. Self‐monitoring of blood glucose levels is also recommended in some but not all cases.^8^ Managing this complex regimen is demanding for everyone with type 2 diabetes, but living with an SMI presents additional challenges.^9^ A systematic review of adherence to diabetes medication in people with SMI reported adherence rates between 51% and 85%.^10^ A study in East London^11^ found that people with diabetes and SMI were more likely to smoke and to be obese and less likely to have had retinopathy screening than those without SMI, and target blood glucose level was achieved by less than half of participants.

To help enhance diabetes self‐management among people with SMI, it is first of all important to understand the challenges they face, but to date, there has been only one study of which we are aware,^12^ and no research in UK, that has asked people with diabetes and SMI about their diabetes self‐management. The aim of this study was to identify the barriers and enablers to effective diabetes self‐management experienced by people with SMI.

## 2 METHODS

This is a qualitative study using semi‐structured interviews informed by theories of behaviour change.

Participants were recruited via community mental health teams (CMHT) in an inner London borough. One of the research team (FLG) presented the study to the clinical teams in each CMHT. Of the four CMHTs that were contacted, all agreed to participate in the study. Eligible participants were recruited from three CMHTs. Service users were eligible for the study if they were aged 18 years and over, had a diagnosis of type 2 diabetes and SMI (defined as schizophrenia, schizoaffective disorder, bipolar disorder, personality disorder or depression with psychotic features), were able to speak English and were considered by their care co‐ordinator or psychiatrist —based on a clinical team review in consultation with the psychiatrist and wider multidisciplinary team— to be well enough to give consent to take part in an interview. The interviewer also asked volunteers to verbalize their understanding of the research when taking consent, prior to commencing the interview, to ensure capacity. Recruitment continued until saturation was reached, that is when no new themes relating to the research question emerged in the interviews.^13^

Information about the study was sent or given to eligible service users by their care co‐ordinator or psychiatrist, and details of those who expressed an interest in taking part were passed to the research team to arrange a convenient time for interview. Interviews were conducted in a private room in the community mental health service. Participants were interviewed by FLG or MH. Interviews were audio‐recorded with participants' permission and transcribed verbatim. Participants were each given £20 for their time and expenses.

The interview topic guide was developed in collaboration with an established research advisory group of mental health service users and carers^14^ and people with diabetes. The guide was designed to explore how participants managed their diabetes and the barriers or enablers to successful diabetes self‐management. It was based on the Theoretical Domains Framework (TDF),^15^ which was developed from a synthesis of 33 behaviour change theories and consists of 14 theoretical domains that have been found to influence behaviour. The domains are 1) knowledge, 2) skills, 3) social/professional role and identity, 4) beliefs about capabilities, 5) optimism, 6) beliefs about consequences, 7) reinforcement, 8) intentions, 9) goals, 10) memory, attention and decision processes, 11) environment context and resources, 12) social influences, 13) emotion and 14) behavioural regulation. A brief description of the domains is included in the interview topic guide in Appendix A. This framework was used to help ensure that the interviews would capture a comprehensive range of potential factors that could act as barriers or enablers of participants' diabetes self‐management behaviour. The interview topic guide (see Appendix A) included questions based on each of these 14 domains but was semi‐structured, allowing the interviewer and interviewee flexibility to explore issues in further detail.

### 2.1 Ethical approval

The study received ethical approval from the National Health Service (NHS) Health Research Authority Proportionate Review Sub‐Committee of the National Research Ethics Service Committee South Central—Oxford C. REC Reference 14/SC/0274. Approval was also obtained from the Research and Development office of the NHS Trust where the research was conducted. Participants provided written informed consent before taking part in the interview.

### 2.2 Analysis

Data analysis was informed by the TDF and followed four steps:

1. Two of the authors (FG and KM) independently coded participant responses into the 14 theoretical domains of the TDF. They then compared coding and discussed any differences to reach consensus, consulting with a third author (HM) if necessary.
2. “Belief statements” were generated to represent collections of responses with similar underlying meanings. Belief statements were created by KM and checked by FG with any queries discussed with HM.
3. The frequency of each belief statement, that is the number of participants who expressed a particular belief statement, was calculated.
4. The most relevant belief statements within each domain were identified through consensus discussion in the research team which comprises researchers in long‐term conditions (KM, HM), mental health (AS, MH, JJ, CF), health services research (FLG) and a service user (DT). Relevant beliefs were selected on the basis of the following established criteria^16^, ^17^: a) frequency, b) the presence of conflicting beliefs, for example “I know/don't know how to manage my diabetes,” which could be a barrier to diabetes self‐management for some but an enabler for others, c) perceived salience of the belief as likely to impact on diabetes self‐management behaviour d) had been reported as important in the literature.

Relevance was judged by considering the four criteria concurrently so for example, a belief with lower frequency could be relevant if it appeared to be important in impacting on diabetes self‐management behaviour.

To check that the belief statements represented the relevant domains, an independent researcher, who is experienced in using the TDF and was blind to the original coding, mapped the belief statements back onto the domains.

## 3 RESULTS

A total of 21 service users were screened for participation. Of these, five were excluded due to the following: insufficient English (n=1), being too unwell (n=3), or about to move to another service (n=1), a further two refused participation. The remaining 14 service users took part in an interview. Participant characteristics are reported in Table 1. Most were male and from Black, Asian and Minority Ethnic (BAME) groups. The most frequent diagnosis was schizophrenia. Interviews lasted between 16 and 46 minutes, median 25 minutes. Precise date of diagnosis of diabetes and/or SMI was not available for all participants, but duration of SMI ranged from approximately one to 50 years, median seven years, and duration of diabetes ranged from ten months to approximately twenty years, median 6 years.

**Table 1:** Participant characteristics (n=14)

Characteristic	
Age in years, mean (SD)	47 (14)
Gender, n (%) male	9 (64)
Ethnicity, n (%)	
Black African Caribbean	5 (36)
South Asian	5 (36)
White	1 (7)
Mixed White/African Caribbean	3 (21)
Mental health diagnosis, n (%)	
Schizophrenia	7 (50)
Schizoaffective disorder & schizophrenia	1 (7)
Bipolar disorder	3 (21)
Personality disorder	1 (7)
Depression with psychotic features	2 (14)
Duration of SMI in years, median (range)	7 (1‐50)
Self‐reported duration of diabetes, median (range)	6 years (10 mo‐20 y)
Diabetes treatment, n (%)	
Insulin	3 (21)
Oral medication	10 (71)
Lifestyle	1 (7)

All TDF domains were mentioned by at least four of the 14 participants. The most relevant beliefs within each theoretical domain are summarized below, organized within their relevant theoretical domains. Table 2 gives sample quotes for each of these beliefs.

**Table 2:** Specific beliefs

Specific beliefs within each domain	Sample quotes	No. of participants who expressed belief
Behavioural regulation		14
I have a routine for taking my diabetes medication	Well when I first started taking them I had to write down when I had to take it, but I know, I've got an automatic clock, I know when to take it, and yeah. (SU2)	8
I find it difficult to establish a routine to manage my diabetes	And then at lunchtime instead of eating I'll have to go to the gym instead, and it's, it just, sometimes it just doesn't fit in (SU3) I still have bad sleeping patterns and eating patterns (SU1)	2
Beliefs about capabilities		13
I am confident that I can manage my diabetes	From my point of view I'm confident I can manage. (SU7)	8
I find it difficult to control my sugar intake	Is the sugar, that's all, craving the sugar, sugar drinks and the gateau (SU10)	7
Beliefs about consequences		14
If I don't manage my diabetes properly I will have poor health	If your blood glucose levels go high then immediately your main organs will get affected, and then you're going to pay the price aren't you? (SU6)	11
If I don't manage my diabetes properly it will make my mental health worse	I think mood swings are caused by glucose levels (SU6)	4
Emotion		13
If my mental health is poor I find it difficult to manage my diabetes	Sometimes my moods as well, like when I'm hyper I'm very, what's the word, lackadaisical like I just don't care or […] So I could go when I'm hyper I could go maybe like two, three days without eating and then I'll binge eat (SU4)	9
Managing my diabetes worries me	Really scared now, I don't know is, how to control it better. (SU10)	6
Environmental context and resources		13
I have access to the health services I need to help me manage my diabetes	What health services are available to help you manage your diabetes [names health centres] Is it ever difficult to see them or is it OK? Do you manage to make appointments? They send me appointments and I attend them regularly. (SU13)	5
The available diabetes education does not meet my needs	Yeah, I think the book that is for the diabetes monitoring, for the BM machine, you know the little book where it says before lunch, before breakfast and that sort of thing, I don't know if that can change to something very, very simple (SU12)	2
Goals		13
I want to control my diabetes to protect my health	I know the importance of it right now, OK? If I feel that in the future I will continue to do the same thing because I know in the long run it's my health and at the same time I would like to feel good, you know I would like to feel good. (SU5)	7
I want to control my diabetes so that I can do the things I want to in life	I want to feel good, and when you feel good you can do many good things. (SU6)	5
Managing my diabetes and my mental health are equally important	It all depends on how I manage my moods really, because it seems like one feeds into the other, if your mood's crap then you don't look after yourself and then your diabetes takes a toll on you physically. When you feel physically like crap it takes a toll on your mood, so they both feed into each other, so it's like trying to find a positive way of managing the two. (SU3)	4
Intentions		12
I intend to take steps to manage my diabetes in the future	Yeah, I will, I'll take the tablets unless the doctor says oh, you're OK now (SU2) And in terms of food I've completely ruled out junk food now, whatever I want to eat I will make sure I buy fresh and cook at home and I'll do it on my own. (SU6) I try the best I can to find a way to deal with it.(SU8)	12
I do not intend to manage (some aspect of) my diabetes in the future	Do you, did you ever, were you ever given things to measure your own blood sugar level with? Oh yeah, I have got all the mechanism, but I'm going to chuck them I think. (SU2) I think about [attending blood tests], but I procrastinate a lot.(SU1)	4
Knowledge		9
I know about diabetes and its possible complications	Diabetes I know pretty good, I know what it is, it's a very, very terrible disease. (SU8) They did, without sounding funny, they do a lot of scare tactics, their little booklet shows people with like half a foot and (SU4)	4
I know how to manage my diabetes	And I know what kind of foods can be bad for me (SU1)	4 (4)
I don't know how to manage my diabetes	The main thing is up to now they haven't been giving me proper guidance about diabetes […] They did say don't do this, don't do this, don't do that, so. They haven't said how to manage it. (SU7)	3
Memory, attention and decision processes		13
Managing my diabetes is confusing	Because you know diabetes how, is, if you done one day mistake with the medication, or your, all diabetes are going up and down, and you can't understand what's going on. (SU11)	5
Remembering all the things I need to do to manage my diabetes is difficult	I forget about diabetes and I will be thinking something else and then forgot about what I should eat and I shouldn't eat. (SU7)	4
Remembering all the things I need to do to manage my diabetes is not difficult	Yeah, I never forgot to use my medicine (SU8)	5
Optimism		8
I am [somewhat] optimistic that I will be able to manage my diabetes in future	Yeah, I think it'll just get better and better with time. (SU1) I might be able to, I'll learn to leave too much sweet stuff alone. I might be able to manage it. (SU14)	6
I am not optimistic that I will be able to manage my diabetes in future	It does make me not to be very optimistic about saying that I won't be able to really manage it the way I want to. (SU8)	3
Reinforcement		4
Seeing the benefits of managing my diabetes is rewarding	Good man, it feels very good. Yes tell me I look man, I'm managing well, I'm managing that well. (SU5)	3
Skills		9
Managing diabetes (does not) require(s) particular skills	Do you think there are any special skills or techniques to help you manage your diabetes? Not really. (SU10)	3
Social influences		14
My friends and family help me to manage my diabetes	My mum's been really supportive, and when she nags and stuff, even when she's not around and I'm making decisions, I can hear her in my head. (SU1)	11
My GP and practice nurse help me to manage my diabetes	The first person I will talk to about it is my doctor, my GP (SU8)	11
My mental health team help me to manage my diabetes	When I told [my care coordinator] that my blood sugars were at 30 he was like, […], you need to seriously get on top of your medication and get tested on a daily basis, and for the next week I'm going to be phoning you every day for the numbers. And so because I knew that he was going to be on my backside I was a bit, because I had to hold myself accountable I was a bit more on the point with it (SU3)	9
My diabetes specialists help me to manage my diabetes	And I know what kind of foods can be bad for me and also learnt that from, it was the diabetic nurse from the place I was last living, and I used to have to see her a bit frequently. Did she come and see you or did you have appointments like at the clinic to see her or I made appointments to go and see her, maybe once a week or once every couple of weeks, and she would tell me stuff like that. She would give me lots of advice on how to keep my blood sugars down and the things that I needed to do, she'd check my feet and stuff like that. (SU1)	8
Social professional role & identity		13
Managing my diabetes is my responsibility	Yeah, it's up to me. It's up to me to manage it. It's my responsibility. It's my duty. It's nobody's, but I have to make certain, because people don't always remember, but I have got to remember. (SU5)	12

Bold values are the interviewer words.

### 3.1 Knowledge

Participants were aware of the risk of diabetes complications including amputation, blindness, kidney damage and heart disease. They were also aware of the need to take medication for their diabetes where prescribed and to eat a healthy diet, and most expressed awareness that they should keep physically active. The need to maintain a healthy weight was expressed by six participants. In spite of a general awareness of the need to eat healthily, participants mainly focused on a need to reduce sugar/carbohydrate intake and some gaps in knowledge were apparent, for example aiming to reduce sugar intake by cutting out fizzy drinks, but replacing them with fruit juice, which also has a high sugar content, or thinking that not eating much sugar could lead to blood glucose levels falling dangerously low.

Participants were also aware of the need to have their blood glucose levels checked, and the three participants who were taking insulin all self‐monitored their blood glucose. Self‐monitoring of blood glucose levels is not routinely offered to all adults with type 2 diabetes,^8^ and most of those who were taking oral medication were not self‐monitoring, but had blood tests taken at their GP practice.

The need for foot care was not reported spontaneously, and when asked specifically, only one participant was performing regular foot checks. Most participants were aware of the need to attend retinopathy screening.

A perceived lack of knowledge of diabetes or its management was rarely given as a barrier to diabetes self‐management although one participant did make the point that he had received conflicting dietary advice from different health‐care teams.

### 3.2 Skills

Most participants did not identify any particular skills that were necessary to manage diabetes, but one did report that being able to cook meals rather than buy ready meals was important.

### 3.3 Memory, attention and decision processes

Five participants reported never forgetting to take their medication, but half of the participants reported finding management of aspects of their diabetes confusing and/or forgetting to perform some self‐management behaviours, particularly if they were feeling mentally unwell. For three people, this meant forgetting to take their medication and possibly repeating doses; of these, one also reported forgetting about what he should or should not eat. Another participant, who was taking insulin, reported forgetting to check their blood glucose levels. Five participants reported finding diabetes management confusing, for example not understanding particular aspects of self‐management such as blood glucose monitoring or dietary advice or why fluctuations in blood glucose had occurred. Of these, one reported hearing voices that interfered with his ability to make appropriate decisions about his diabetes self‐management. The responses of this participant and of one other sometimes appeared quite confused, which may also reflect difficulties in Memory, Attention and Decision Processes.

### 3.4 Behavioural regulation

Although five participants were self‐monitoring their blood glucose levels, only one person reported adjusting their food based on these readings. It was unclear whether others were using this information to inform their diabetes management. Seven participants had developed a routine for taking their medication, although two of them reported being inconsistent in keeping to this routine.

Two people reported keeping to a regular exercise routine, and three had a plan for how they managed their diet. This included preparing a healthy packed lunch for work, eating small portions and avoiding eating late in the day. Most participants, however, had not developed plans for how to manage their diabetes and two spoke of having poor or chaotic eating patterns.

### 3.5 Social professional role & identity

Almost all participants felt that it was their responsibility to manage their diabetes; only one participant discussed the difficulty she had had in accepting the diagnosis, which had influenced her diabetes self‐management. This had, however, improved as she became more accepting of her diabetes.

### 3.6 Goals

Participants recognized the importance of managing their diabetes, and only one person reported a lack of motivation to do so. Others though recognized the impact that becoming mentally unwell had on their motivation.

The diabetes self‐management behaviours reported as the most important were taking medication and eating healthily. Participants hoped that managing their diabetes effectively would help protect their health and general well‐being and enable them to do the things they want to in life, such as being there for their families. Although the issue of prioritizing mental health or diabetes was generally not raised by participants, four people reported giving equal priority to the management of their mental health and diabetes. Weight loss was a goal for one person although several were overweight.

### 3.7 Intention

Although participants had reported the importance of managing their diabetes, four acknowledged that they did not always intend to follow a healthy lifestyle or to attend recommended health checks. For example, one participant put off attending appointments and another did not intend to use the blood glucose monitor he had been given. Another reported that he would not take medication if he sensed that there was still diabetes medication in his system and that he did not need it. One participant who had a diagnosis of personality disorder reported that there had been instances when she had felt suicidal and intended to self‐harm by allowing her blood glucose levels to run dangerously high. No other participant reported an intention to use their diabetes in this way.

The most commonly expressed behavioural intention was to eat a healthy diet and/or cut down on unhealthy foods; however, it was often very clear that participants did not manage to keep to this intention.

### 3.8 Beliefs about Consequences

Most participants believed that if they did not manage their diabetes properly, it would have adverse health consequences, which could include amputation, coma, heart attack, blindness, stroke, kidney damage, infection or death. Four participants also believed that failure to manage their diabetes properly would adversely affect their mental health.

### 3.9 Beliefs about capabilities

Although eight participants reported feeling confident that they could manage their diabetes, difficulties in managing diabetes were reported. Taking diabetes medication was not perceived as difficult by most participants, but keeping to a healthy diet, particularly controlling sugar intake, was reported as very challenging by half of participants who spoke of difficulty resisting cravings for sweet foods and drinks.

### 3.10 Emotion

Almost all participants reported that their emotions had an influence on their diabetes self‐management. When feeling mentally unwell, nine participants reported being unable to manage and of neglecting their diabetes including not taking their medication. Five people recognized a relationship between their mood and their ability to eat healthily, which included comfort eating when mood was low or going for long periods without food and binge eating when experiencing high moods.

Managing different aspects of their diabetes was a worry for six participants, for example not being able to identify the hidden sugars in foods, concern about interactions between their different diabetes and psychoactive medications, being scared about not being able to better control diabetes, fear of going into a coma and worrying about possible deterioration that may have occurred between infrequent diabetes health checks.

### 3.11 Reinforcement

Only four participants identified reinforcement as a factor that influenced their diabetes self‐management; of these, two people reported that they found it rewarding to manage their diabetes successfully.

### 3.12 Optimism

Six participants reported being optimistic about managing their diabetes in the future; however, levels of optimism varied and most were fairly qualified in their optimism. For example, some reported that they would be able to keep their diabetes under control as long as they were able to keep taking their medication or manage their mental health or gain control over their eating or as long as they received help. Unrealistic optimism was reflected in the responses of at least one participant who felt that his diabetes would get better with time even though he also reported being overweight, eating too much unhealthy food, needing to exercise more and not attending appointments or retinopathy screening.

### 3.13 Social Influence

Support for managing their diabetes was recognized as important by almost all participants. Helpful support from family and friends included reminders to take medication, changing the family diet and encouragement to exercise and eat healthily. However, not everyone had friends or family they could rely on, and five people reported that family and friends did not get involved or that they did not need their support.

Participants reported receiving help and advice with managing their diabetes from a range of health professionals. GPs were generally perceived as supportive in carrying out diabetes checks and providing medication, but two participants reported having a poor relationship with their GP or practice nurse, and another did not regularly attend appointments. Advice about how to manage diabetes had been received by all participants, mostly from practice nurses or diabetes specialist nurses, but six participants reported not following the advice they received or not accepting the support offered.

Mental health professionals were considered an important source of diabetes support by nine participants, for example by checking blood glucose levels, providing advice or reminding them to attend appointments. However, five participants reported that their mental health team focused only on their mental health and were not involved in their diabetes management.

### 3.14 Environmental context and resources

Environmental factors that were perceived to negatively impact on self‐management were reported by five participants and included poor financial status; not being provided with blood glucose monitoring equipment and difficulty managing diabetes around work or social occasions. Four participants reported finding Internet resources helpful for managing their diabetes.

Accessing health services for diabetes was generally not reported as a difficulty; however, five participants expressed some concerns, which included infrequent check‐ups, the need for better holistic care or co‐ordination between mental health and diabetes, and that the diabetes education received did not meet their needs.

## 4 DISCUSSION

To our knowledge, this is the first UK study that has asked people with SMI about the factors that affect the management of their diabetes. Use of a theoretical framework to develop our interview enabled us to identify a broad range of potential barriers and enablers to diabetes self‐management. Important potential enablers included that participants saw it as their role to manage their diabetes and expressed a goal or intention to try to do so. Where participants were able to establish and maintain a routine, it was perceived as helpful. Family and health professionals were seen as an important source of support for managing diabetes. A key barrier to managing diabetes was poor mental health, due to either low mood or cognitive difficulties.

Most participants considered self‐management of their diabetes to be important, saw it as their responsibility and were very aware of the risks of complications if they did not manage their diabetes successfully. They were also largely aware of the diabetes self‐management behaviours that were required. In spite of this, several were not physically active, did not carry out regular foot checks and struggled to eat a healthy diet.

Previous research has found poorer levels of diabetes knowledge in those with SMI.^18^ Although the current study did identify some misperceptions, overall participants were aware of the risks of diabetes complications and the self‐management behaviours required to achieve good diabetes control. Nam^19^ conducted a systematic review of barriers to diabetes management in the general diabetes population and concluded that knowledge alone did not necessarily result in good adherence to recommended self‐management behaviours. Our findings in a sample of people with SMI support this conclusion. It would seem that messages from health professionals regarding the potential adverse consequences of failure to control diabetes have been received and understood; however, other approaches are needed to help people with SMI successfully change their behaviour to better manage their diabetes.

Participants were very aware of the consequences of not managing diabetes successfully, and whilst this did appear to have the effect of people wanting to avoid diabetes complications, it may also be a factor in participants' lack of optimism about their future diabetes control. This may be an important focus for interventions aimed at people with SMI and type 2 diabetes as hope and optimism for the future have been identified as important concepts in the facilitation of recovery in people with SMI^20^ and may potentially impact on other health behaviours. There may be a complex interaction between hope and recovery with an important role for social context and interpersonal relationships, including those with clinicians who occupy a powerful position in relation to service users' hope^21^ and recovery.^22^

We identified important barriers that would need to be addressed to help improve self‐management; these included how people manage diabetes when they are experiencing instability in their mental health, such as mood disturbances or psychotic phenomena. Despite its importance, there is limited evidence concerning self‐management interventions for people with diabetes and SMI. Although there is emerging and promising evidence for self‐management approaches for a range of long‐term medical conditions and SMI,^23^ few high‐quality evaluations have been conducted for SMI and diabetes. A recent Cochrane review^24^ identified a single study^25^, ^26^ that showed benefits from a lifestyle intervention for diabetes and weight loss. The Cochrane review concluded that there is a need for theoretical and evidence‐based self‐management interventions to help people with SMI to manage their diabetes.

This study identified that although some participants had managed to form routines which they found helpful for managing their diabetes, particularly for taking medication, development of plans to help diabetes self‐management more broadly was uncommon. Although most participants had a goal or expressed an intention to eat healthily, many found it difficult to do so, a problem which is also experienced in the broader diabetes population.^27^ The difficulty of translating intentions into behaviour is also well recognized.^28^ Self‐regulatory processes such as forming coping and action plans^29^ or implementation intentions (“if‐then” plans for how to act in given situations) have been found to help bridge the gap between intentions and behaviour,^29^, ^30^ and recent research suggests that their use may have potential for improving outcomes for people with SMI.^31^, ^32^ Once mental health deteriorates significantly, people with SMI are unable to manage their diabetes, but incorporating simple plans or implementation intentions into care plans may help them to make diabetes self‐management part of a regular routine and to identify and take action to combat early signs of deteriorating mental health. With recent evidence suggesting the effectiveness of Wellness Recovery Action Plans (WRAP) in people with SMI,^33^ incorporating diabetes management within WRAPs may offer a beneficial way forward.

The support of family and friends, where it was available, was perceived as an important enabler of diabetes self‐management for many. Involving families and carers in the care and support of people with SMI can provide numerous benefits,^34^ and identifying effective ways to draw on lay sources of support may also have potential for helping people with SMI with their diabetes self‐management. There is some evidence for the benefits of peer support in diabetes,^35^ but definitions and mode of support vary considerably and findings are inconsistent.^36^ A trial of diabetes self‐management for people with SMI using trained peer educators is currently underway in USA.^37^

Several participants reported that their GP was their first port of call for managing their diabetes, primarily conducting health checks, but others acknowledged and valued the important role that could be played by their mental health team. This support was mostly in terms of being reminded to attend appointments and encouragement to live healthily. There was variability, however, in participants' experiences of support for diabetes self‐management from their mental health team, and in some cases, they reported that mental health professionals did not address physical health issues. Role ambiguity^38^ and diagnostic overshadowing^39^ have been found among mental health nurses regarding the provision of physical health care, but this study has highlighted that mental health professionals have a potentially important role in promoting diabetes self‐management. Poor awareness of national guidelines for managing diabetes has, however, been reported by mental health professionals.^40^ Given the generally low levels of attendance at structured diabetes education classes^41^, ^42^ and the impact of mental health on diabetes self‐management reported by participants, training and supervision of mental health professionals to promote self‐management in people with type 2 diabetes and SMI is essential.

The study has a number of limitations. Diabetes self‐management involves several behaviours, and the barriers and enablers may vary across these behaviours. To explore this issue in more depth would have required repetition of the interview questions for each individual behaviour, but this would have been too burdensome for participants. We therefore asked about diabetes self‐management in general.

The people who took part in an interview may not have been those with the greatest barriers to engaging with health services so we may have interviewed those who are more actively engaged in managing their diabetes.

Interviews were conducted with a small number of participants of predominantly African Caribbean and South Asian origin from a single mental health service, which may limit transferability of the findings. The London borough from which participants were recruited has a high proportion of BAME residents, which is reflected in our sample. In the 2011 Census,^43^ 44% of residents described themselves as Asian/Asian British, 29% as White and 20% as Black/Black British. Risk of developing type 2 diabetes is higher in people from South Asian and Black communities,^44^ which also helps to explain our sample characteristics. We did, however, exclude non‐English speakers, whose experiences of negotiating the health service may be different from the participants in our study. A number of reviews have examined cultural influences on diabetes self‐management,^45^, ^46^ and an in‐depth discussion of the issue is not within the scope of this study. However, Wilson et al^46^ concluded that there was little evidence for substantial differences in self‐management behaviours between ethnic groups, although there may be distinct dietary pressures that interventions developed to help enhance diabetes self‐management should take into account.

This study identified several factors that may be important to support people with SMI to manage their diabetes, but further research is required to investigate whether these factors predict performance of self‐management behaviours. A survey of people with SMI and type 2 diabetes is currently underway to examine how these theoretically informed factors affect diabetes self‐management in a larger population.

In conclusion, this study found that people with type 2 diabetes and SMI considered it important to manage their diabetes but often struggled to do so effectively. Tailored interventions are required that can help people with SMI to manage the additional challenges presented by living with a comorbid diagnosis of type 2 diabetes. Mental health professionals have an important role to play in supporting diabetes self‐management in their patients.

## CONFLICTS OF INTEREST

None.

## APPENDIX A

### A.1

#### Introduction

Thank you for agreeing to take part in this interview. We are conducting this study to help us understand how people with mental illness manage their diabetes, what helps them and what they find difficult so that we find ways of helping people in the future. Some of the questions may appear to be quite similar or obvious to answer but we are trying to understand the topic from different perspectives so bear with me, take your time and answer as frankly as possible.

Are you happy to get started?

I'd like to start by asking you to tell me about when you first found out you had diabetes.

1. **Knowledge** (An awareness of the existence of something)*.

What kind of things do you need to do to manage your diabetes? Prompt: monitoring own blood sugar, taking medication, checking feet, having eye checks, healthy diet, healthy weight, physical activity, stress management; are there any checks you do yourself or get done by a health professional?

How would you rate these in order of importance? Why?

How does this change when you are unwell?

What information or education have you received about your diabetes? Who gave you this information? When?

1. **Skills** (An ability or proficiency acquired through practice.)

Are there any skills or techniques that help you manage your diabetes? Prompt: In relation to….monitoring blood sugar, taking medication, checking feet, having eye checks, healthy diet (shopping, budgeting), healthy weight, physical activity

1. **Social/professional role and identity** (A coherent set of behaviours and displayed personal qualities of an individual in a social or work setting.)

As someone living with both diabetes and mental illness do you feel it is up to you to manage your diabetes? Why?

Is there anyone else who should be involved? Which health professionals help you to manage your diabetes? What do they do?

How does managing diabetes fit in with your life on a day‐to‐day basis?

1. **Beliefs about capabilities** (Acceptance of the truth, reality, or validity about an ability, talent, or facility that a person can put to constructive use.)

What do you find most challenging about managing your diabetes? Is there anything you struggle with?

How confident are you that you can manage your diabetes?

1. **Optimism** (The confidence that things will happen for the best or that desired goals will be attained.)

How optimistic are you that in the future you will be able to manage your diabetes? What makes you optimistic / not optimistic?

1. **Beliefs about consequences** (Acceptance of the truth, reality, or validity about outcomes of a behaviour in a given situation.)

What do you think would happen if you didn't look after your diabetes?

1. **Reinforcement** (Increasing the probability of a response by arranging a dependent relationship, or contingency, between the response and a given stimulus.)

What encourages you to manage your diabetes?

What discourages/stops you from managing your diabetes?

1. **Intentions** (A conscious decision to perform a behaviour or a resolve to act in a certain way.)

How do you think you will manage your diabetes in the future?

What would happen when you are unwell?

1. **Goals** (Mental representations of outcomes or end states that an individual wants to achieve.)

When you think about all the things you have to do every day, how important is managing your diabetes?

1. **Memory, attention and decision processes** (The ability to retain information, focus selectively on aspects of the environment and choose between two or more alternatives.)

Is managing your diabetes something you have to give a lot of thought to?

Are decisions about how you manage your diabetes easy or difficult to make? In what way?

1. **Environmental context and resources** (The ability to retain information, focus selectively on aspects of the environment and choose between two or more alternatives.)

Are there times or situations in the past when you have had problems and not been able to manage your diabetes? Prompts: time constraints, getting to clinic

What health services are available to help you manage your diabetes? Prompts: Is it difficult to get to see them? What difficulties have you had? What would make it easier?

1. **Social influences** (Those interpersonal processes that can cause individuals to change their thoughts, feelings, or behaviours.)

If you were having a problem with your diabetes, who would you turn to?

What support does the health service offer for managing your diabetes? What support would you like from the health service?

How do your friends or family help you to manage your diabetes?

In what ways do they make it easier or harder for you to manage your diabetes? Prompt: what you eat, exercising, taking you to appointment, reminding you about monitoring you diabetes or taking medication

1. **Emotion** (A complex reaction pattern, involving experiential, behavioural, and physiological elements, by which the individual attempts to deal with a personally significant matter or event.)

In what way does how you feel emotionally affect whether you can manage your diabetes?

1. **Behavioural regulation** (Anything aimed at managing or changing objectively observed or measured actions.)

How is managing your diabetes covered in your care plan?

If you wanted to change the way you managed your diabetes how would you do this? Can you think of a recent example of this? What did you do differently?

Do you make specific plans to help you manage your diabetes? Can you tell me what they are?

What would help you manage your diabetes better in future?

**Close**: That's all the questions I have for you, has anything occurred to you about this topic during the interview that we haven't discussed?

Thank you for taking part.

*Definitions of domains from Cane et al. 15.
